# The mechanisms of late-onset synaptic responses in a realistic model of Unipolar Brush Cells

**DOI:** 10.1186/1471-2202-14-S1-P79

**Published:** 2013-07-08

**Authors:** Sathyaa Subramaniyam, Paola Perin, Francesca Locatelli, Sergio Masetto, Sergio Solinas, Egidio D'Angelo

**Affiliations:** 1 Department of Brain and Behavioural Science, Neurophysiology and Neurocomputation Unit, University of Pavia, Via Forlanini 6, I-27100, Pavia, Italy; 2 Consorzio Interuniversitario per le Scienze Fisiche della Materia (CNISM), Via Bassi 6, I-27100 Pavia, Italy; 3 Brain Connectivity Center, Istituto Neurologico IRCCS C. Mondino, Via Mondino 2, I-27100 Pavia, Italy

## 

Unipolar brush cells (UBCs) are excitatory glutamatergic interneurons of the cerebellar granular layer receiving both primary and secondary vestibular inputs through mossy fibers (excitatory input) and Golgi cell axon (inhibitory input). When injected with progressively increasing depolarizing currents from a negative membrane potential, the UBC generates a burst sustained by a calcium spike and then a protracted discharge with shorter latency and spike frequency adaptation. The intrinsic excitability of UBCs is determined by an H current and by Low Voltage activated and High Voltage activated calcium currents [2,3]. Fast inactivating T-type Calcium channels generate low-threshold spikes and L-type Calcium channel sustain tonic firing. The H current (activated between -60 mV and -80 mV) produces a slow hyperpolarization characterized by a "sag" in response to a hyperpolarizing step and an afterhyperpolarization at the end of a depolarizing step. Here we present a biologically realistic multi-compartmental mathematical model of the UBC realized with the NEURON-PYTHON simulator. According to literature [1-4], ionic channels are distributed among compartments (soma, dendrite, initial segment and axon). The model can reproduce the excitable properties of UBCs in current-clamp and voltage-clamp modes. The response to mossy fiber inputs was reproduced using synaptic models of AMPA and NMDA synaptic receptors. The model is also capable of reproducing the late onset response recently reported for this cellular type [5] by exploiting the interaction between cAMP, TRPC, and H current. This model, in addition to confirm the primary role of the aforementioned currents in UBC's electroresponsiveness, will prove a valuable tool for investigating the UBC's function in the cerebellar network.
